# Adaptation to Collao Quechua and psychometric analysis of the instrument for detecting violence against women

**DOI:** 10.17843/rpmesp.2025.422.14426

**Published:** 2025-06-12

**Authors:** Esmeralda Calsina-Rosa, Ruth Huaycani-Cotrado, Evelyn Magaly Yucra-Ticona, Julio Cjuno

**Affiliations:** 1 Peruvian Union University, Professional School of Psychology, Juliaca, Peru. Peruvian Union University Peruvian Union University Professional School of Psychology Juliaca Peru; 2 César Vallejo University, School of Medicine, Piura, Peru. César Vallejo University César Vallejo University School of Medicine Piura Peru

**Keywords:** Intimate partner violence, indigenous peoples, psychometrics, battered women

## Abstract

**Objectives.:**

To determine the validity, measurement invariance, and reliability of the Women’s Abuse Screening Tool (WAST) in the Collao Quechua language for Peruvian women.

**Materials and methods.:**

A psychometric study was conducted to adapt the WAST to the Collao Quechua language of Puno and Cusco in a non-probability sample of 521 women, 46.1% of whom were between the ages of 18 and 34. Initially, the WAST was directly and reverse translated, then five expert judges reviewed the Collao Quechua version, and a focus group of women confirmed the clarity and comprehensibility of the items. Multigroup confirmatory factor analysis (CFA) was used for measurement invariance, reliability analysis, and external validity using Spearman’s rho.

**Results.:**

The unidimensional model of the Collao Quechua WAST reported adequate goodness-of-fit values (CFI= 0.995; TLI= 0.992; SRMR=0.051; RMSEA=0.063) and showed measurement invariance by age, educational level, place of residence, and monthly family income (Δ CFI or Δ RMSEA < 0.01). In terms of external validity, the Collao Quechua WAST and the Patient Health Questionnaire (PHQ-9) showed a direct relationship with moderate strength (Rho=0.618, p=0.001); it also reported optimal reliability, α =0.860 and ω=0.867.

**Conclusions.:**

The unidimensional Collao Quechua WAST showed validity of its internal structure, external validity, invariance by age, educational level, place of residence, and monthly family income, and optimal reliability. Its use is recommended for screening for intimate partner violence in women who speak Collao Quechua.

## INTRODUCTION

The World Health Organization reported in 2021 that approximately one in three women worldwide have experienced physical and/or sexual violence by their partner ^1^, while in Latin America and the Caribbean in 2022, 4,050 women were reported as victims of femicide ^2^. In Peru in 2020, it was reported that 54.8% of women were victims of violence by their spouse ^3^. Particularly in regions of the Peruvian Andes such as Puno, Cusco, and others where the Quechua people live, estimates show that 62.5% of women in indigenous communities have experienced intimate partner violence ^4^.

Intimate partner violence refers to behaviors by a partner or former partner that cause physical, sexual, or psychological harm to the victim ^5^; it is usually caused by problems in the relationship dynamic and reinforced by violent behaviors or non-assertive communication based on the breakdown of patterns of good behavior ^6^. It has been found that women with low levels of education, who suffered violence in childhood, those who have a partner who consumes alcohol, have had an abortion, and those who have rigid and traditional parental stereotypes are more likely to be victims ^7^. Social reinforcement theory explains that intimate partner violence can occur due to the presence of a social model that generates an imitative pattern in observers ^8^. The historical anthropology of intimate partner violence explains that it could have been generated from the very existence of human beings, since machismo and the practice of patriarchy have been present since the earliest societies ^9^. On the other hand, the ecological model maintains that violent behavior is determined by personal traits, interaction with the surrounding environment, gender differences, context, and is also influenced by beliefs, lifestyles, and cultural habits ^9^. In light of this, it is important to have tools for screening intimate partner violence in order to identify cases of intimate partner violence at an early stage.

The Woman Abuse Screening Tool (WAST) is a unidimensional instrument for screening indicators of intimate partner violence that is widely preferred due to its evidence of validity and reliability ^10^. It has been validated in different parts of the world since its initial construction in English for adult women in the United States, where it showed adequate validity, as the construct explained 85% of the variance of the intimate partner violence construct and had a reliability of 0.95 ^11^. In Japan, a version was adapted that reported sensitivity between 66.7% and 71.4%, as well as a specificity of 89.7% ^12^. In Iran, the WAST was validated in Persian, demonstrating 93% sensitivity and 71% specificity in primary health care screening ^13^. In Indonesia, the WAST was adapted to Hindi, with a sensitivity of 84.9% and a specificity of 61.0% with a reliability of α=0.80, reporting optimal reliability with a cutoff point of 10 points for the presence of intimate partner violence ^14^. Likewise, the WAST has been adapted to French, with a sensitivity of 97.7%, a specificity of 97.1%, and a reliability of 0.95, demonstrating adequate values for use in primary health care ^15^. It was also adapted to Greek, with this version reporting a sensitivity of 99.7% and a specificity of 64.4%, as well as a reliability of 0.92 using the classic alpha ^16^.

Validation studies of the WAST in Peruvian Spanish have been conducted in female university students; this study reported adequate evidence of validity of its internal structure for a unidimensional model (GFI=0.997; CFI=0.994; TLI=0.992; SRMR=0.049; RMSEA=0.058), as well as its reliability α=0.86 and ω=0.90 ^17^. Recently, the WAST was validated in older Peruvian women with an average age of 35 years from rural and urban areas. This study reports that the goodness-of-fit indices are adequate for a unidimensional model (CFI=0.998; TLI=0.991; RMSEA=0.103; SRMR=0.075). Furthermore, the reliability coefficients (α=0.899 and ω=0.916) reported optimal values for use in Peruvian women ^18^.

However, there are 55 indigenous communities in Peru, and the Quechua are the largest indigenous community, with 3,735,682 (13.9%) of Peruvians speaking Quechua ^19^. Since this is a community with a language, culture, beliefs, and lifestyle that differ from the general population, it is necessary to have a screening tool for intimate partner violence that is adapted to their context. Therefore, this study aimed to determine the validity and reliability of the Collao Quechua version of the Women’s Abuse Screening Tool (WAST) in Quechua-speaking Peruvian women.

**KEY MESSAGES**

Motivation for the study: Intimate partner violence is a serious public health problem, with higher prevalence in indigenous communities. Therefore, it is essential to have culturally and linguistically appropriate tools that allow for early screening of this form of violence among Quechua-speaking women.

Main findings: The Women’s Abuse Screening Tool (WAST) adapted to Collao Quechua showed evidence of internal and external validity, optimal reliability, and invariance of measurement according to age, educational level, place of residence, and monthly family income in a sample of Quechua-speaking women.

Implications: The Collao Quechua WAST can be implemented as a screening tool in primary health care services and in state institutions that address cases of intimate partner violence in regions where Collao Quechua is spoken, promoting early detection and timely response to this problem.

## MATERIALS AND METHODS

### Design and context

This was a cross-sectional psychometric study that aimed to determine the validity of the internal structure and reliability of the Women’s Abuse Screening Tool (WAST) in its Collao Quechua version for Peruvian women ^20^.

The process of cultural adaptation was carried out in three stages. The first stage consisted of the direct translation process; initially, two translators participated, who individually translated the items and response options from Peruvian Spanish into Collao Quechua (direct translation); after that, the translators and the research team met to consolidate and compare the translations in order to reach a consensus on a single version of the Collao Quechua WAST. Next, the reverse translation from Collao Quechua to Peruvian Spanish was carried out by two other translators who worked independently. The translators and researchers then met to compare the differences and similarities between the original Spanish version and the translated version from Collao Quechua to Spanish, and finally approved the final version. All four translators were bilingual, read and spoke Peruvian Spanish and Collao Quechua, and had professional training in Quechua linguistics or translation.

The second stage consisted of adapting the Collao Quechua WAST according to the criteria of expert judges, with the participation of five Quechua-speaking psychologists with between one and five years of work experience in caring for women victims of intimate partner violence in Quechua-speaking communities in Puno and Cusco. The items of the Collao Quechua WAST were presented to the experts in a digital form in Microsoft Word via the WhatsApp social network; the experts reviewed the clarity, relevance, and representativeness of the items in a total of one to two rounds of review until achieving an Aiken V coefficient score in which the lower limit of the 95% confidence interval (95% CI) was greater than 0.70 (the 95% CI of Aiken V was between 0.76 and 1.00) in the three content validity indicators.

After that, a focus group was organized with the participation of a Quechua-speaking psychologist with experience in qualitative interviews as moderator and a group of thirteen Collao Quechua-speaking women (eight from Cusco and five from Puno), who gave their informed consent prior to their participation. The focus group was held via a Zoom meeting in which the moderator initially shared a URL for the online survey and asked all participants to complete the Collao Quechua WAST. Next, using two questions, 1) Did you find any questions that you did not understand or were difficult to understand? and 2) Did you find any words in Quechua that you had never heard before or that were new to you? we determined the clarity of understanding the instrument items based on the participants’ responses. Once the findings were recorded as written notes and the recommendations were implemented, the final version of the Collao Quechua WAST was approved (available at: https://doi.org/10.5281/zenodo.15226607).

### Participants

The study involved 521 adult speakers of the Collao variety of Quechua, which is spoken in the departments of Cusco and Puno, Peru. A non-probability convenience sample was used, due to the capabilities of the research team, while the sample size was estimated using a calculator (https://wnarifin.shinyapps.io/ss_sem_cfi_equal/); although it is usual to use a minimum power of 80%, in this study we used a power of 95% to ensure greater accuracy in the estimation of parameters and minimize the risk of type II error and an expected dropout rate of 5%, one dimension and eight items expecting a minimum expected sample (n=365). Women over the age of 18 who gave their free and voluntary consent to participate in the study were included. They also had to be able to read at least the Collao variety of Quechua in addition to Spanish (bilingual) and live in any city or rural area in the departments of Puno or Cusco. On the other hand, women who spoke a different variety of Quechua, who partially responded to the survey, and who had any temporary or permanent mental or physical condition that prevented them from responding coherently to the survey questions were excluded.

### Instruments

The instrument to be adapted is called the Woman Abuse Screening Tool (WAST) and has been translated and adapted to the Peruvian context for use with adult women and women studying at Peruvian universities ^17^^,^^18^. This instrument has reported a single factor with eight items that have Likert-type response options (1 = never, 2 = sometimes, and 3 = many times). Based on the sum of the direct scores of the items, scores between 8 and 24 points can be obtained, with higher scores indicating a higher indicator of intimate partner violence. This instrument, in its Peruvian Spanish version, has reported adequate adjustments (CFI=0.998; TLI=0.991; RMSEA=0.103; SRMR=0.075), which indicate internal structure validity and solid evidence of reliability (α=0.899 and ω=0.916).

Likewise, for external validity, we used the Patient Health Questionnaire (PHQ-9) in Collao Quechua adapted for Peruvian adults who speak Quechua ^21^. This instrument measures depressive symptoms based on the last two weeks, with a unidimensional model consisting of nine items and Likert-type responses ranging from “Mana hayk’aqpas” = 0 to “Yaqa llapa p’unchawkuna” = 3, from which direct scores of 0 to 27 points can be calculated. In terms of validity, it has reported adequate goodness-of-fit indices (CFI=0.99; TLI=0.99, RMSEA=0.058, SRMR=0.042) as well as optimal evidence of reliability (α=0.915; ω=0.881).

### Procedures

Once approved by the Ethics Committee, we provided training on the data collection method, and data collection was planned in the cities of Juliaca and Cusco. The survey was administered by three interviewers using a digital Google form. The initial section presented the informed consent form; only individuals who gave their consent were allowed to proceed to answer the printed questionnaire or URL sent to each participant so that it could be filled out on their cell phone; the interviewers remained with the participants until they finished and submitted their responses. Data collection was carried out door-to-door and in parks of these cities, initially asking whether they spoke Quechua from Puno or Cusco, beginning in the first week of June and ending at the end of July 2024.

### Statistical analysis

We used descriptive statistics, such as absolute and relative frequency, for characterization variables. Likewise, we estimated Aiken’s V coefficient for the items, considering them valid when the lower limit of the 95% confidence interval was ≥0.70.

In a subsequent phase, given that all versions of the WAST have always shown one-dimensionality ^22^, was used the Confirmatory Factor Analysis (CFA) with the WLSMV (Weighted Least Square Mean and Variance Adjusted) estimator, since the instrument has ordinal response options and response options with fewer than five categories ^23^. The analysis reported the standard measures of goodness of fit: X^2^ for model versus baseline, considering values <3 acceptable; the comparative fit index (CFI), which is adequate when >0.90; and the Tucker-Lewis Index (TLI), which is acceptable when >0.90. Likewise, the standardized root mean square residual (SRMR) and the root mean square error of approximation (RMSEA) were considered adequate with values ≤0.08 ^24^. Subsequently, we used the graphical analysis of Structural Equation Modeling (SEM) in order to determine the factor loadings of the items against the latent factors and their respective unexplained variances.

Likewise, we analyzed measurement invariance using the multigroup CFA method, considering groups defined by age, educational level, place of residence, and monthly family income. The difference in CFI (ΔCFI) or RMSEA (ΔRMSEA) was the criterion for determining the models with more restrictions versus those with fewer restrictions, initially assuming configural invariance as the base model, scaling to metric, strong, and strict invariance. In order to determine an adequately restricted and appropriate model at each stage of invariance, the ΔCFI or ΔRMSEA had to have values <0.01 ^25^.

On the other hand, we used the Spearman’s Rho correlation coefficient to determine validity based on the relationship with other variables, considering external validity when Rho> 0.50. Likewise, McDonald’s Omega coefficient was used to estimate reliability, considering it reliable when it had a value ≥ 0.70 ^26^. All analyses were performed in the statistical program R Studio version 2025.05.0+496 using the Lavaan, SemTools, and SemPlot packages.

### Ethical considerations

This research was approved by Report No. 2024-CEB-FCS - UPeU-107 by the Ethics Committee of the Universidad Peruana Unión in Juliaca. It also complied with all ethical principles of research involving human subjects, such as autonomy through the use of informed consent for participants ^27^.

## RESULTS

The sample consisted of 521 participants, most of whom were women between the ages of 18 and 34, with a total of 240 (46.1%). We found that 217 (41.7%) women had secondary education, 321 (61.6%) lived in urban areas, and 313 (60.1%) had a monthly income of less than 1,025 soles per month (Table 1).

**Table 1:** Characteristics of the study population (N=521).

Characteristics		n	(%)
Age			
	18 to 34 years	240	(46.1)
	35 to 54 years	219	(42.0)
	55 years or more	62	(11.9)
Level of education			
	Primary school or less	116	(22.3)
	Secondary school	217	(41.6)
	Higher education	188	(36.1)
Household			
	Rural	200	(38.4)
	Urban	321	(61.6)
Monthly income			
	Less or equal than S/. 1025.00	313	(60.1)
	More than S/. 1025.00	208	(39.9)

The content validity estimated by Aiken’s V coefficient ranged between 0.86 and 1.00 for the three criteria (relevance, representativeness, and clarity) for the eight items of the Collao Quechua WAST. All items had lower 95% CI limits greater than 0.70, indicating adequate content validity (Table 2).

**Table 2:** Aiken’s V coefficient for items from the Woman Abuse Screening Tool (WAST) in Collao Quechua.

	Relevance		Representativeness		Clarity	
	V	(95%CI)	V	(95%CI)	V	(95%CI)
Item 1	0.93	(0.76 - 0.98)	0.93	(0.76 - 0.98)	0.93	(0.76 - 0.98)
Item 2	0.93	(0.76 - 0.98)	1.00	(0.86 - 1.00)	1.00	(0.86 - 1.00)
Item 3	1.00	(0.86 - 1.00)	0.93	(0.76 - 0.98)	0.93	(0.76 - 0.98)
Item 4	1.00	(0.86 - 1.00)	1.00	(0.86 - 1.00)	0.93	(0.76 - 0.98)
Item 5	1.00	(0.86 - 1.00)	1.00	(0.86 - 1.00)	1.00	(0.86 - 1.00)
Item 6	0.93	(0.76 - 0.98)	0.93	(0.76 - 0.98)	0.93	(0.76 - 0.98)
Item 7	0.93	(0.76 - 0.98)	0.93	(0.76 - 0.98)	0.93	(0.76 - 0.98)
Item 8	1.00	(0.86 - 1.00)	1.00	(0.86 - 1.00)	1.00	(0.86 - 1.00)

When performing confirmatory factor analysis, we found that the single-factor model reported adequate goodness-of-fit values for the Collao Quechua WAST (CFI=0.995; TLI=0.992; SRMR=0.051; RMSEA=0.063) (Table 3).

**Table 3:** Confirmatory factor analysis of the Woman Abuse Screening Tool (WAST) in Collao Quechua.

Goodness of Fit Index	WAST (n=521)
X² (df)	61.319
CFI	0.995
TLI	0.992
RMSEA	0.063
95%CI	0.045 - 0.081
SRMR	0.051

X^2^ (df) for the model versus the baseline, CFI: Comparative fit index, TLI: Tucker-Lewis’s index, SRMR: Standardized root mean squared residual, RMSEA: Root mean squared error of approximation, 95%CI: 95% confidence interval.

**Figure 1:**
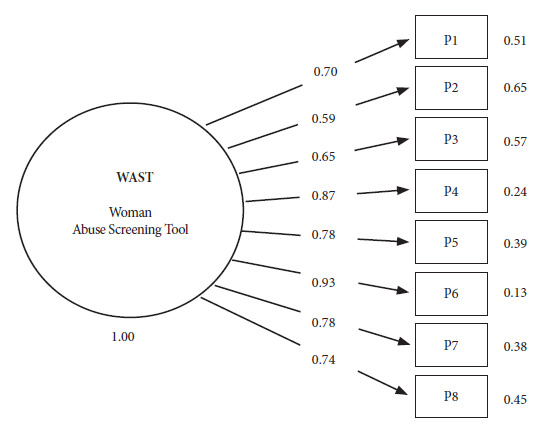
Structural equation modeling of the Woman Abuse Screening Tool (WAST) in Collao Quechua.

By applying Structural Equation Modeling (SEM) we obtained factor loadings with a minimum of λ=0.59 and a maximum of λ=0.93 in the items of the Collao Quechua WAST (Figure 1).

The results of the multigroup AFC confirm the invariance of measurement according to age, level of education, place of residence, and monthly family income, given that the configurational, metric, and strict model presented adequate goodness-of-fit indices, considering that the CFI presented values >0.95 and the RMSEA values <0.08, with the exception of metric invariance in monthly family income (RMSEA=0.090). Additionally, the absolute values Δ CFI and/or Δ RMSEA reported values <0.01 at all levels of invariance (Table 4).

**Table 4:** Measurement invariance through multigroup confirmatory factor analysis of the WAST instrument in Collao Quechua.

Grouping variable	Invariance (Model)	X^2^	gl	p	CFI	Δ CFI	RMSEA	Δ RMSEA
Age	1. Configural	113.253	60	<0.001	0.993	-	0.071	-
	2. Metrical	186.456	74	1.127	0.985	-0.007	0.089	0.022
	3. Strong	145.811	88	1.057	0.992	0.006	0.061	-0.032
	4. Strict	145.811	88	1.057	0.992	0.001	0.061	0.001
Education	1. Configural	99.732	60	9.727	0.993	-	0.061	-
	2. Metrical	151.165	74	3.157	0.987	-0.006	0.077	0.015
	3. Strong	148.527	88	5.877	0.989	0.002	0.063	-0.014
	4. Strict	148.527	88	5.877	0.989	0.001	0.063	0.001
Place of residence	1. Configural	73.985	40	8.621	0.995	-	0.057	-
	2. Metrical	142.442	47	1.529	0.986	-0.008	0.088	0.031
	3. Strong	128.466	54	5.226	0.989	0.002	0.072	-0.015
	4. Strict	128.466	54	5.226	0.989	0.001	0.072	0.001
Monthly family income	1. Configural	78.102	40	2.941	0.994	-	0.060	-
	2. Metrical	146.154	47	4.217	0.986	-0.008	0.090	0.029
	3. Strong	176.947	54	5.440	0.982	-0.003	0.089	0.003
	4. Strict	176.947	54	5.44	0.982	0.001	0.089	0.001

X² (gl): Chi-square statistic with degrees of freedom, CFI: Comparative Fit Index, TLI: Tucker-Lewis Index, SRMR: Standardized Root Mean Square Residual, RMSEA: Root Mean Square Error of Approximation, Δ: difference.

Regarding external validity based on the relationship with another variable, we found that the overall score of the Collao Quechua WAST and the Patient Health Questionnaire (PHQ-9) show a direct, highly significant relationship and a moderate strength of relationship (Rho=0.618, p=0.001); this confirms external validity. Likewise, the unidimensional model of the Collao Quechua WAST reported good reliability, with Cronbach’s alpha values of 0.860 and McDonald’s omega of 0.867.

## DISCUSSION

In a sample of 521 bilingual Quechua-speaking women, most of whom were aged between 18 and 34, had completed secondary education, lived in urban areas, and had a monthly household income of less than 1,025 soles, the Collao Quechua WAST with a unidimensional model showed adequate goodness-of-fit indices, guaranteeing the validity of its internal structure. On the other hand, external validity was also adequate when comparing the WAST and PHQ-9 scores in their Collao Quechua versions. It also showed optimal reliability and was invariant across age groups, educational level, place of residence, and monthly household income.

The single-factor model of the Collao Quechua WAST reported adequate fits, suggesting that it is a good model for assessing intimate partner violence as a single factor. Fit indices such as the CFI (0.995) and TLI (0.992) indicate an excellent fit of the model, as both values are very close to 1. The SRMR (0.051) is also within the acceptability parameter (<0.08), suggesting a good representation of the observed data, while the RMSEA (0.063) is below the threshold of 0.08, indicating a reasonable fit of the model ^28^. These results together demonstrate that the single-factor model of the Collao Quechua WAST is robust and adequate, supporting its internal structure validity for measuring intimate partner violence among Quechua-speaking women. In fact, previous studies in young women ^17^^)^ and adult women ^18^^)^ also reported similar goodness-of-fit indices, confirming that this instrument is valid for the Peruvian population.

Our study showed that the Collao Quechua WAST presented measurement invariance among groups defined by age, educational level, place of residence, and monthly family income. Similarly, the Spanish version of the WAST validated in Peruvian women also showed invariance in terms of age, educational level, place of residence, and employment status ^18^. However, the analysis of metric invariance by monthly family income level showed an RMSEA value of 0.090, slightly higher than the recommended threshold (<0.08). This result could be influenced by the unequal or small size of some subsamples within this sociodemographic variable, which may affect the stability of the model estimates and artificially increase the approximation error indices. It is important to note that RMSEA is particularly sensitive to models with low degrees of freedom or small sample sizes in subgroups, which can lead to an overestimation of the fit error even in correctly specified models. Despite this slightly higher value, simulation studies have suggested that RMSEA values up to 0.10 can be considered acceptable under certain conditions, especially when other indices such as the CFI remain within appropriate ranges ^29^. Similarly, the previous study about the Spanish version of the WAST in the Peruvian population reported similar RMSEA values from invariance analyses ^18^, which reinforces the robustness of our findings.

The overall score of the Collao Quechua WAST and the Patient Health Questionnaire (PHQ-9) showed a direct, highly significant relationship and a moderate strength of relationship (Rho=0.618, p=0.001). Another study also reported similar results, with the Peruvian Spanish version of the WAST showing a correlation with the PHQ-9 score (Rho=0.620; p=0.001). This finding indicates that as WAST Quechua Collao scores increase, so do PHQ-9 scores. On the other hand, the moderate strength of the relationship (Rho=0.618) suggests a considerable association between the two measures, and the highly significant p-value (p=0.001) confirms that the observed correlation is highly unlikely to occur by chance, thus solidly confirming criterion validity or validity based on the relationship with other variables, indicating that it is a valid tool for assessing intimate partner violence in clinical and research settings. The reason for choosing to relate it to a test that assesses depression to evaluate external validity was due to the strong association between intimate partner violence and depressive symptoms documented by several studies ^30^^,^^31^.

The Collao Quechua WAST reported good reliability, with Cronbach’s alpha values of 0.860 and McDonald’s omega of 0.867. These findings were similar to those found in the Peruvian version for Spanish-speaking adult women, where the reliability coefficients α = 0.899 and ω = 0.916 also reported optimal values ^18^. This indicates that the Collao Quechua WAST shows solid evidence of accuracy and consistency in measurement at different times for this population. Given that reliability is understood as a characteristic of the scores of an instrument that analyzes the accuracy of measurements at different times and is mainly related to random error, where the greater the random error, the lower the reliability, despite the questioning of the use of Cronbach’s alpha due to limitations based on the number of items, response alternatives, and proportion of variance ^26^, it presented a coefficient within the optimal reliability parameter, as did McDonald’s omega coefficient, which, unlike the classic alpha, estimates reliability based on factor loadings, making calculations more stable and reflecting a real reliability value ^32^.

The WAST items are used in mental health screening in primary health care and government programs to identify early indicators of intimate partner violence. The Collao Quechua WAST could be implemented in this system for Quechua-speaking populations of the Collao variety spoken in southern Peru. Future assessments could therefore better address the real problem of intimate partner violence in indigenous populations.

Our study has several limitations, such as the fact that it is validated in women with schooling who can read Quechua, meaning that women without schooling could not be evaluated with this written version. Subsequent studies could design an audio version that reaches these unschooled populations. On the other hand, although significant correlations were found between the PHQ-9 and WAST in Collao Quechua, these should not be interpreted as evidence of causality, since our instrument may have high external validity in this specific population but not necessarily in others. Therefore, these findings must be generalized with caution to other Quechua-speaking populations or in different cultural contexts. Another limitation is the possible presence of social desirability bias, which limits the accuracy in detecting actual cases. Although anonymity was guaranteed, it cannot be ruled out that some participants underestimated their experience with intimate partner violence due to cultural norms or fear of stigma. In addition, another relevant limitation is that the Collao Quechua WAST assesses intimate partner violence from a one-dimensional perspective. Although this feature allows for rapid and general detection of the problem, it also limits its ability to accurately identify the specific nature of the experienced violence. Consequently, while the instrument is useful for initial screening, it does not replace more detailed assessments by professionals that allow for the characterization of different types of violence. The main strength of our study is that it is the first intimate partner violence instrument adapted to the cultural context of Quechua Collao that will serve to identify early and more accurately cases of intimate partner violence that are overlooked due to cultural and linguistic barriers.

In conclusion, the WAST in Collao Quechua, based on a unidimensional model, demonstrated internal validity, external validity, optimal reliability, and was found to be invariant across age groups, educational level, place of residence, and monthly family income among Peruvian women. Given these findings, we recommended its use for the early identification of cases of intimate partner violence among women who speak Collao Quechua.
