# Supplementary material for: Adaptation to Collao Quechua and psychometric analysis of the instrument for detecting violence against women
Source: Rev Peru Med Exp Salud Publica. 2025 Jun 12;42(2):175–83. doi: 10.17843/rpmesp.2025.422.14426 (PMC12377885; doi:10.17843/rpmesp.2025.422.14426)
Supplement: Supplementary material. — Available in the electronic version of the RPMESP. [file rpmesp-42-02-14426-s001.docx]

**ANEXOS**

**ANEXO 1**

**WAST Quechua Collao**

| No | Tapukuykuna | Ñishu k'irinchasqa  *Mucha tensión* | Pisi k'irinchasqa  *Algo de tensión* | Mana k’irinchasqa  *Sin tensión* |
| --- | --- | --- | --- | --- |
| 1 | ¿Llapampi, imaynatan qan yuyaychariwaq qosaykiwan kausayñiykita?  *En general, ¿cómo describiría usted su relación de pareja?* |  |  |  |
|  |  | Ñishu sasachaywan  *Mucha dificultad* | Pisi sasachaywan  *Algo de dificultad* | Manan sasachu  *Sin dificultad* |
| 2 | ¿Imayna sasachaywanmi qan qosaykiwan chaninchanki otaq allchanki sasachayñiykista?  *¿Con cuanta dificultad usted y su pareja resuelven sus diferencias?* |  |  |  |
|  |  | Sapa Kutin  *Muchas veces* | Pisi Kutillan  *A veces* | Mana hayk'aqpas  *Nunca* |
| 3 | ¿Llapa huchatarisqakismanta qan llakisqacho otaq k'irisqachu kashanki qanwan?  *¿Al terminar las discusiones usted se siente decaída o mal con usted misma?* |  |  |  |
| 4 | ¿Llapa huchatariy tukukun k'irinakuywanchu, hayt'akuywanchu icha t'anqarpariywanchu?  *¿Las discusiones terminan en golpes, patadas o empujones?* |  |  |  |
| 5 | ¿Sinchi mancharisqa kanki yanaykiq ima rimarisqanwan otaq imapis ruwarunasunkimanta?  *¿Usted se siente aterrada por lo que su pareja pueda decir o hacer?* |  |  |  |
| 6 | ¿Hayk'aqllapas qosayki maqarasunkichu qanta?  *¿Alguna vez su pareja ha abusado fisicamente de usted?* |  |  |  |
| 7 | ¿Hayk'aqllapas qosayki siminchaspa K'araqta millayta k'amirasunki otaq ch'aqwaspa k'irirasunki qanta?  *¿Alguna vez su pareja ha abusado emocionalmente de usted?* |  |  |  |
| 8 | ¿Hayk'aqllapas qosayki mana munashaqtiyki sat'irasunkichu/violarasunkichu* qanta?  *¿Alguna vez su pareja ha abusado sexualmente de usted?* |  |  |  |

**ANEXO 2**

**GLOSARIO**

| Español | Quechua Collao | Adecuación |
| --- | --- | --- |
| Relación de pareja | Qosaykiwan kausayñiyki | Tu vida con tu esposo |
| Mucha tensión | Ñishu k’irinchasqa | Demasiado tenso |
| Algo de tensión | Pisi k’irinchasqa | Un poco tenso |
| Sin tensión | Mana k’irinchasqa | Sin tensión |
| Mucha dificultad | Ñishu sasachaywan | Mucha dificultad |
| Algo de dificultad | Pisi sasachaywan | Un poco de dificultad |
| Sin dificultad | Manan sasachu | Sin dificultad |
| Muchas veces | Sapa kutin | Frecuentemente |
| A veces | Pisi kutillan | A veces |
| Nunca | Mana hayk’aqpas | Nunca |
| Decaída / mal con usted misma | Llakisqacho / k’irisqachu kashanki | Triste o mal consigo misma |
| Golpes, patadas o empujones | K’irinakuy, hayt’akuy, t’anqarpariy | Acciones de violencia física |
| Aterrada | Sinchi mancharisqa | Muy asustada |
| Lo que su pareja diga o haga | Ima rimarisqanwan otaq imapis ruwarunasunkimanta | Dichos o acciones de la pareja |
| Abuso físico | Maqarasunkichu | Te ha golpeado |
| Abuso emocional | Millayta k’amirasunki / Ch’aqwaspa k’irirasunki | Insultos o maltrato verbal |
| Abuso sexual | Mana munashaqtiyki sat’irasunkichu / violarasunkichu | Forzada sexualmente |
