# Supplementary material for: Adaptation to Collao Quechua and psychometric analysis of the instrument for detecting violence against women
Source: Rev Peru Med Exp Salud Publica. 2025 Jun 12;42(2):175–83. doi: 10.17843/rpmesp.2025.422.14426 (PMC12377885; doi:10.17843/rpmesp.2025.422.14426)
Supplement: Translation

# Translation: Adaptación al quechua Collao y análisis psicométrico del instrumento para la detección de violencia contra la mujer

**Authors:** Esmeralda Calsina-Rosa, Ruth Huaycani-Cotrado, Evelyn Magaly Yucra-Ticona, Julio Cjuno

**Affiliations:** 1 Universidad Peruana Unión, Escuela Profesional de Psicología, Juliaca, Perú.; 2 Universidad César Vallejo, Escuela de Medicina, Piura, Perú.

**Keywords:** Violencia de pareja, pueblos indígenas, psicometría, mujeres maltratadas

## Abstract

**Objetivos:**

. Determinar la validez, invarianza de medida y confiabilidad del instrumento para la detección de violencia contra la mujer (WAST) versión en quechua Collao para mujeres peruanas.

**Material y métodos.:**

Estudio psicométrico, que buscó adaptar al quechua Collao de Puno y Cusco en una muestra no probabilística de 521 mujeres donde el 46,1% tienen entre 18 a 34 años. Inicialmente se realizó la traducción directa e inversa, luego cinco jueces expertos revisaron la versión en quechua Collao y un grupo focal de mujeres confirmó la claridad y comprensión de los ítems. Se empleó el Análisis Factorial Confirmatorio (AFC) multigrupos para la invarianza de medida, análisis de confiabilidad y validez externa empleando el Rho de Spearman.

**Resultados:**

. El modelo unidimensional del WAST quechua Collao reportó adecuados valores de bondad de ajuste (CFI= 0,995; TLI= 0,992; SRMR=0,051; RMSEA=0,063), presentó invarianza de medida por edad, grado de instrucción, lugar de vivienda e ingreso económico familiar mensual (Δ CFI o Δ RMSEA < 0,01). En cuanto a la validez externa el WAST quechua Collao y el Patient Health Questionnaire (PHQ-9) presentaron una relación directa con fuerza moderada (Rho=0,618, p=0,001); asimismo, reportó una óptima confiabilidad, α =0,860 y ω=0,867.

**Conclusiones:**

. El WAST quechua Collao unidimensional presentó validez de su estructura interna, validez externa, invarianza por edad, grado de instrucción, lugar de vivienda e ingreso económico familiar mensual y óptima confiabilidad. Su uso está recomendado para el tamizaje de la violencia de pareja en mujeres que hablan quechua Collao.

## INTRODUCCIÓN

La Organización Mundial de la Salud en el 2021 refiere que, alrededor de una de cada tres de las mujeres en el mundo han sido sometidas a violencia física y/o sexual por su pareja ^1^, mientras que en América Latina y el Caribe en el 2022 se reportó 4 050 mujeres fueron víctimas de feminicidio ^2^; en Perú en el 2020 se ha reportado que el 54,8% de mujeres fueron víctimas de violencia por su cónyuge ^3^. Particularmente en regiones de los andes peruanos como Puno, Cusco y otros donde habitan los quechuas se estimó que el 62,5% de las mujeres de comunidades originarias han experimentado violencia de pareja ^4^.

La violencia de pareja son aquellos comportamientos de la pareja o expareja que generan un daño físico, sexual o psicológico en la víctima ^5^; usualmente está originado por problemas en la dinámica relacional y reforzada por conductas violentas o transacciones de comunicación no asertiva basada en la ruptura de los patrones de una buena conducta ^6^. Se ha encontrado que tienen más probabilidad de ser víctimas las mujeres con baja escolaridad, que sufrieron violencia en su infancia, los que tienen una pareja que consume bebidas alcohólicas, sufrieron un aborto y quienes presentan estereotipos parentales rígidos y tradicionales ^7^. La teoría del reforzamiento social explica que la violencia de pareja puede darse debido a la presencia de un modelo social que genera un patrón imitativo en los espectadores ^8^. La antropología histórica de la violencia de pareja explica que podría haberse generado desde la misma existencia del ser humano, puesto que el machismo y la práctica del patriarcado se ha observado desde las primeras sociedades ^9^. Por otro lado, el modelo ecológico sustenta que la conducta violenta es determinada por los rasgos personales, la interacción con el ambiente que lo rodea, las diferencias basadas en el sexo, el contexto y también influenciada por las creencias, estilos de vida y hábitos culturales ^9^. Frente a ello, resulta importante contar con herramientas de tamizaje de violencia de pareja para identificar casos de violencia de pareja de manera precoz.

El instrumento para la detección de violencia contra la mujer y conocido por su nombre en inglés como Woman Abuse Screening Tool (WAST) es un instrumento unidimensional para el tamizaje de los indicadores para violencia de pareja ampliamente preferido por sus evidencias de validez y fiabilidad ^10^; ha sido validada en diferentes partes del mundo desde su construcción inicial en inglés para mujeres adultas de Estados Unidos donde presentó adecuada validez puesto que el constructo explicaba el 85% de la varianza del constructo de violencia de pareja y presentó una fiabilidad de 0,95 ^11^, en Japón se adaptó una versión que reportó sensibilidad entre 66,7 y 71,4%, así como una especificidad de 89,7% ^12^, en Irán se realizó la validación al idioma persa del WAST, dicho instrumento demostró que era sensible al 93% y su especificidad al 71% en el tamizaje en la atención primaria de la salud ^13^, en Indonesia buscaron adaptar el WAST al idioma hindi, donde el instrumento demostró una sensibilidad de 84,9% y una especificidad de 61,0% con una fiabilidad de α=0,80 que reporta óptima fiabilidad con un punto de corte en 10 puntos para la presencia de violencia de pareja ^14^. Asimismo, el WAST se ha adaptado al francés donde ha reportado una sensibilidad del 97,7%, una especificidad de 97,1% y una fiabilidad de 0,95 demostrando adecuados valores para su uso en la atención primaria de la salud ^15^; también fue adaptado al griego, dicha versión reportó una sensibilidad de 99,7% y una especificidad de 64,4% además de una fiabilidad de 0,92 por el alfa clásico ^16^.

En el contexto del español peruano, se han realizado estudios de validación del WAST en mujeres universitarias; dicho estudio reportó adecuadas evidencias de validez de su estructura interna para un modelo unidimensional (GFI=0,997; CFI=0,994; TLI=0,992; SRMR=0,049; RMSEA=0,058), así como de su fiabilidad α=0,86 y ω=0,90 ^17^ y recientemente se ha encontrado que el WAST fue validado en mujeres adultas peruanas mayores con edad promedio de 35 años de zona rural y urbana, dicho estudio reporta que los índices de bondad de ajuste son adecuados para un modelo unidimensional (CFI=0,998; TLI=0,991; RMSEA=0,103; SRMR=0,075), asimismo los coeficientes de fiabilidad (α=0,899 and ω=0,916) reportaron valores óptimos para su uso en mujeres peruanas ^18^.

Sin embargo, en Perú existen 55 comunidades originarias y los quechuas son la comunidad originaria con mayor población que cuenta con 3 735 682 (13,9%) de peruanos quechua hablantes ^19^. Al tratarse de una comunidad con una lengua, cultura, creencias y estilo de vida distintas a la población general es necesario contar con un instrumento de tamizaje de la violencia de pareja adaptado a dicho contexto. Frente a ello, el objetivo del presente estudio fue determinar la validez y fiabilidad del instrumento para la detección de violencia contra la mujer (WAST) versión en quechua Collao en mujeres peruanas quechua hablantes.

**MENSAJE CLAVE**

Motivación para realizar el estudio: La violencia de pareja constituye un grave problema de salud pública, con mayor prevalencia en comunidades originarias. Ante esta realidad, resulta fundamental disponer de instrumentos cultural y lingüísticamente adecuados que permitan el tamizaje temprano de esta forma de violencia en mujeres quechua hablantes.

Principales hallazgos: El instrumento para la detección de violencia contra la mujer (WAST) adaptado al quechua Collao mostró evidencia de validez interna y externa, confiabilidad óptima, e invarianza de medida según edad, grado de instrucción, lugar de residencia e ingreso económico familiar mensual, en una muestra de mujeres quechua hablantes.

Implicancias: El WAST quechua Collao puede ser implementado como herramienta de tamizaje en los servicios de atención primaria de salud y en instituciones del Estado que abordan casos de violencia de pareja en regiones donde se habla el quechua Collao, favoreciendo la detección precoz y el abordaje oportuno de esta problemática.

## MATERIALES Y MÉTODOS

### Diseño y contexto

El presente estudio es de diseño transversal de tipo psicométrico debido a que busca determinar las evidencias de validez de la estructura interna y fiabilidad del instrumento para la detección de violencia contra la mujer (WAST) en su versión en quechua Collao en mujeres peruanas ^20^.

La adaptación cultural fue desarrollada en tres etapas, la primera consistió en el proceso de traducción directa; inicialmente se contó con la participación de dos traductores quienes tradujeron de manera individual los ítems y opciones de respuesta desde el español peruano al quechua Collao (traducción directa); posterior a ello, los traductores y el equipo de investigación se reunieron para consolidar y comparar las traducciones así llegar a un consenso de una versión única del WAST quechua Collao. En seguida se desarrolló la traducción inversa del quechua Collao al español peruano por otros dos traductores quienes realizaron la traducción de manera individual; posteriormente, los traductores e investigadores se reunieron para comparar las diferencias y similitudes entre la versión original en español con la versión traducida del quechua Collao al español y finalmente aprobaron la versión final. Los cuatro traductores fueron bilingües, leen y hablan en español peruano y quechua Collao, y cuentan con formación profesional de lingüista quechua o traducción.

La segunda etapa consistió en la adaptación del WAST quechua Collao por criterio de jueces expertos, que contó con la participación de cinco psicólogos quechua hablantes con experiencia laboral entre uno a cinco años en la atención de mujeres víctimas de violencia de pareja en comunidades quechua hablantes de Puno y Cusco. Los ítems de WAST quechua Collao fueron presentados a los expertos en un formulario digital en Microsoft Word por medio de la red social WhatsApp; los expertos revisaron la claridad, relevancia y representatividad de los ítems en un total de uno a dos rondas de revisión hasta lograr puntaje del coeficiente V de Aiken en el que el límite inferior del intervalo de confianza al 95% (IC 95%) fuera mayor a 0,70 (el IC95% del V de Aiken fue entre 0,76 a 1,00) en los tres indicadores de validez de contenido.

Posterior a ello, se organizó un grupo focal con la participación de un psicólogo quechua hablante con experiencia en entrevistas cualitativas como moderador y un grupo de trece mujeres quechua hablantes de la variedad Collao (ocho de Cusco y cinco de Puno), quienes antes de su participación brindaron su consentimiento informado. El grupo focal fue desarrollado en una sala Zoom en el que inicialmente el moderador compartió un URL de la encuesta virtual y solicitó que todas las participantes puedan responder el WAST quechua Collao. En seguida, empleando dos preguntas 1) ¿Encontraron alguna pregunta que no se entendió o era difícil de entender? y 2) ¿Encontraron alguna palabra en quechua que nunca escucharon o es nuevo para ustedes? Se determino la claridad de comprensión de los ítems del instrumento a partir de las respuestas de las participantes. Toda vez registrado los alcances en apuntes escritos e implementado las recomendaciones se aprobó la versión final del WAST quechua Collao (Disponible en: https://doi.org/10.5281/zenodo.15226607).

### Participantes

Participaron en el estudio 521 adultos quechua hablantes de la variedad Collao que es hablado en los departamentos de Cusco y Puno, Perú. Se empleó un muestreo no probabilístico por conveniencia, debido a las posibilidades del equipo de investigación; mientras que el tamaño de muestra fue estimado empleando una calculadora (https://wnarifin.shinyapps.io/ss_sem_cfi_equal/); aunque lo usual es emplear una potencia mínima del 80%, en nuestro estudio empleamos una potencia del 95% buscando asegurar una mayor precisión en la estimación de parámetros y minimizar el riesgo de error de tipo II y una tasa de abandono esperado del 5%, una dimensión y ocho ítems esperando una muestra mínima esperada (n=365). Se incluyeron a mujeres mayores de 18 años, que brindaron su consentimiento de forma libre y voluntaria para participar en el estudio, además mínimamente podían leer en quechua de la variedad Collao además del español (bilingües), así también deben estar viviendo en cualquier ciudad o zona rural de los departamentos de Puno o Cusco. Por otro lado, se excluyeron a mujeres que hablaban otra variedad del quechua, quienes respondieron parcialmente la encuesta y presentaron alguna condición mental o física que sea temporal o permanente que impida responder con coherencia las preguntas de la encuesta.

### Instrumentos

El instrumento para adaptar se denomina Detección de Violencia Contra la Mujer conocido por su nombre en inglés Woman Abuse Screening Tool (WAST) y actualmente se encuentra traducida y adaptada al contexto peruano y su aplicación en mujeres adultas y mujeres que estudian en universidades peruanas ^17^^,^^18^. Dicho instrumento ha reportado un solo factor con ocho ítems que tienen opciones de respuesta tipo Likert siendo (1= nunca, 2= a veces y 3= muchas veces) a partir de la sumatoria de la puntuación directa de sus ítems se pueden obtener puntuaciones entre 8 y 24 puntos considerando que a mayor puntaje mayor es el indicador de violencia de pareja. Este instrumento en su versión en español peruano ha reportado adecuados ajustes (CFI=0,998; TLI=0,991; RMSEA=0,103; SRMR=0,075) que declaran una validez de estructura interna y sólidas evidencias de fiabilidad (α=0,899 y ω=0,916).

Asimismo, para la validez externa se utilizó la Patient Health Questionnaire (PHQ-9) en quechua Collao adaptado en adultos peruanos quechua hablantes ^21^. Dicho instrumento mide los síntomas depresivos en función a las últimas dos semanas, con un modelo unidimensional conformado por 9 ítems y respuesta tipo Likert que van desde “Mana hayk’aqpas”= 0 a “Yaqa llapa p’unchawkuna” = 3, a partir de ello se pueden calcular puntajes directos de 0 a 27 puntos. En cuanto a la validez ha reportado adecuados índices de bondad de ajuste (CFI=0,99; TLI=0,99, RMSEA=0,058, SRMR=0,042) así como óptimas evidencias de fiabilidad (α=0,915; ω=0,881).

### Procedimientos

Toda vez aprobado por el Comité de Ética, se procedió a capacitar sobre el método de recolección de los datos y en seguida se planificó la recolección en las ciudades de Juliaca y Cusco. La encuesta fue administrada por tres encuestadoras mediante un formulario digital de Google. En la sección inicial se presentó el consentimiento informado; únicamente los individuos que otorgaron su consentimiento pudieron proceder a responder el cuestionario impreso o URL a cada participante para que pueda ser llenado desde su celular; las encuestadoras permanecieron con las participantes hasta que termine y envíe sus respuestas. La recolección de datos se realizó casa por casa y en parques de dichas ciudades consultando inicialmente si hablaban el quechua de Puno o Cusco iniciando en la primera semana del mes de junio y terminando a finales del mes de julio del 2024.

### Análisis estadístico

Inicialmente se calcularon los estadísticos descriptivos para las variables de caracterización como la frecuencia absoluta y relativa. Asimismo, se estimó el coeficiente V de Aiken para los ítems considerando válido cuando el límite inferior del intervalo de confianza al 95% fue ≥0,70.

En una siguiente fase, debido a que el WAST en todas sus versiones ha mostrado siempre unidimensionalidad ^22^, se desarrolló el Análisis Factorial Confirmatorio (AFC), utilizando el estimador WLSMV (Weighted Least Square Mean and Variance Adjusted) puesto que el instrumento cuenta con opciones de respuesta ordinal y cuenta con opciones de respuesta menores de 5 categorías ^23^; desde el análisis se reportaron las medidas estándar de bondad de ajuste: el X^2^ para modelo versus línea de base, considerando aceptable valores <3; el índice de ajuste comparativo (CFI), que es adecuado cuando es >0,90; el índice de Tucker-Lewis Index (TLI), que es aceptable cuando es >0,90. Asimismo, el residuo estandarizado cuadrático medio (SRMR) y la raíz del error cuadrático medio de aproximación (RMSEA), considerándolos adecuados con valores ≤0,08 ^24^. Posteriormente se analizó el análisis gráfico de Modelamiento de Ecuaciones Estructurales (SEM) donde se determinaron las cargas factoriales de los ítems frente a los factores latentes y sus respectivas varianzas no explicadas.

Asimismo, se analizó la invarianza de medida por medio del método de AFC multigrupos considerando grupos definidos por edad, grado instrucción, lugar de vivienda e ingreso económica familiar mensual. La diferencia en el CFI (ΔCFI) o RMSEA (ΔRMSEA) fue el criterio para determinar los modelos con más restricciones versus los que tenían menos restricciones, asumiendo inicialmente la invarianza configural como modelo base, escalando a la invarianza métrica, fuerte y estricta. Para poder determinar un modelo adecuadamente restringido y apropiado en cada etapa de invarianza los ΔCFI o ΔRMSEA debieron presentar valores <0,01 ^25^.

Por otro lado, empleando el coeficiente de correlación Rho Spearman se determinó la validez basada en la relación con otras variables considerando validez externa cuando Rho> 0,50. Asimismo, se estimó el coeficiente Omega de McDonald para estimar la confiabilidad considerando confiable cuando presenta un valor ≥ 0,70 ^26^. Todos los análisis se realizaron empleando el programa estadístico R Studio versión 2025.05.0+496 empleando los paquetes Lavaan, SemTools y SemPlot.

### Aspectos éticos

La presente investigación fue aprobada con Informe N° 2024-CEB-FCS - UPeU-107 por el Comité de Ética de la Universidad Peruana Unión sede Juliaca. Asimismo, respetó todos los principios éticos de investigación en seres humanos como la autonomía por el uso del consentimiento informado para los participantes ^27^.

## RESULTADOS

La muestra estuvo conformada por 521 participantes que en su mayoría son mujeres entre 18 a 34 años, con un total de 240 (46,1%); asimismo, la mayor parte de las mujeres cuentan con estudios secundarios siendo 217 (41,7%), por otro lado, 321 (61,6%) radica en el sector urbano, 313 (60,1%) perciben un ingreso económico mensual menor a 1025 soles por mes (Tabla 1).

**Tabla 1:** Características de la población de estudio (N=521).

Características		n	(%)
Edad			
	De 18 a 34 años	240	(46,1)
	De 35 a 54 años	219	(42,0)
	De 55 años a más	62	(11,9)
Grado de Instrucción			
	Primaria o menos	116	(22,3)
	Secundaria	217	(41,6)
	Superior	188	(36,1)
Vivienda			
	Rural	200	(38,4)
	Urbano	321	(61,6)
Ingreso Mensual			
	Menor o igual a S/. 1 025.00	313	(60,1)
	Mayor a S/. 1 025.00	208	(39,9)

La validez de contenido estimado por el coeficiente V de Aiken presentó valores entre 0,86 y 1,00 en los tres criterios (relevancia, representatividad y claridad) para los 8 ítems del WAST quechua Collao. Todos los ítems presentan límites inferiores del IC 95% mayores a 0,70, lo que indica una adecuada validez de contenido (Tabla 2).

**Tabla 2:** Coeficiente V de Aiken de los ítems del Instrumento para la detección de Violencia Contra la Mujer (WAST) en quechua Collao.

	Relevancia		Representatividad		Claridad	
	V	(IC 95%)	V	(IC 95%)	V	(IC 95%)
Ítem 1	0,93	(0,76 - 0,98)	0,93	(0,76 - 0,98)	0,93	(0,76 - 0,98)
Ítem 2	0,93	(0,76 - 0,98)	1,00	(0,86 - 1,00)	1,00	(0,86 - 1,00)
Ítem 3	1,00	(0,86 - 1,00)	0,93	(0,76 - 0,98)	0,93	(0,76 - 0,98)
Ítem 4	1,00	(0,86 - 1,00)	1,00	(0,86 - 1,00)	0,93	(0,76 - 0,98)
Ítem 5	1,00	(0,86 - 1,00)	1,00	(0,86 - 1,00)	1,00	(0,86 - 1,00)
Ítem 6	0,93	(0,76 - 0,98)	0,93	(0,76 - 0,98)	0,93	(0,76 - 0,98)
Ítem 7	0,93	(0,76 - 0,98)	0,93	(0,76 - 0,98)	0,93	(0,76 - 0,98)
Ítem 8	1,00	(0,86 - 1,00)	1,00	(0,86 - 1,00)	1,00	(0,86 - 1,00)

Al realizar el análisis factorial confirmatorio, encontramos que el modelo de un solo factor reportó adecuados valores de bondad de ajuste para el WAST quechua Collao, (CFI=0,995; TLI=0,992; SRMR=0,051; RMSEA=0,063) (Tabla 3).

**Tabla 3:** Análisis factorial confirmatorio del instrumento para la detección de Violencia Contra la Mujer (WAST) en quechua Collao.

Índice de Bondad de Ajuste	WAST (n=521)
X² (df)	61,319
CFI	0,995
TLI	0,992
RMSEA	0,063
IC 95%	0,045 - 0,081
SRMR	0,051

X^2^ (df) para el modelo versus la línea base, CFI: Comparative fit index, TLI: Tucker-Lewis’s index, SRMR: Standardized root mean squared residual, RMSEA: Root mean squared error of approximation, IC 95%: Intervalo de confianza al 95%

Al realizar el Modelamiento de Ecuaciones Estructurales (SEM), se obtuvo cargas de factores con un mínimo de λ=0,59 y un máximo de λ=0,93 en los ítems del WAST quechua Collao (Figura 1).

**Figura 1:** Modelamiento de Ecuaciones Estructurales del instrumento para la detección de Vio-lencia Contra la Mujer (WAST) en quechua Collao.

Los resultados del AFC multigrupos confirma la invarianza de medida según edad, grado de instrucción, lugar de residencia e ingreso económico familiar mensual debido a que el modelo configural, métrica, fuerte y estricta presentaron adecuados índices de bondad se ajuste considerando que el CFI presentó valores >0,95 y el RMSEA valores <0,08 a excepción la invarianza métrica en el ingreso económico familiar mensual (RMSEA=0,090), adicionalmente los valores absolutos Δ CFI y/o Δ RMSEA reportaron valores <0,01 en todos los niveles de invarianza (Tabla 4).

**Tabla 4:** Invarianza de medida por Análisis Factorial Confirmatorio multigrupo del instrumento para la detección de Violencia Contra la Mujer WAST en quechua Collao

Variable de agrupamiento	Invarianza (Modelo)	X2	gl	p	CFI	Δ CFI	RMSEA	Δ RMSEA
Edad	1. Configural	113,253	60	<0,001	0,993	-	0,071	-
	2. Métrica	186,456	74	1,127	0,985	-0,007	0,089	0,022
	3. Fuerte	145,811	88	1,057	0,992	0,006	0,061	-0,032
	4. Estricta	145,811	88	1,057	0,992	0,001	0,061	0,001
Instrucción	1. Configural	99,732	60	9,727	0,993	-	0,061	-
	2. Métrica	151,165	74	3,157	0,987	-0,006	0,077	0,015
	3. Fuerte	148,527	88	5,877	0,989	0,002	0,063	-0,014
	4. Estricta	148,527	88	5,877	0,989	0,001	0,063	0,001
Lugar de vivienda	1. Configural	73,985	40	8,621	0,995	-	0,057	-
	2. Métrica	142,442	47	1,529	0,986	-0,008	0,088	0,031
	3. Fuerte	128,466	54	5,226	0,989	0,002	0,072	-0,015
	4. Estricta	128,466	54	5,226	0,989	0,001	0,072	0,001
Ingreso económico familiar mensual	1. Configural	78,102	40	2,941	0,994	-	0,060	-
	2. Métrica	146,154	47	4,217	0,986	-0,008	0,090	0,029
	3. Fuerte	176,947	54	5,440	0,982	-0,003	0,089	0,003
	4. Estricta	176,947	54	5,44	0,982	0,001	0,089	0,001

X² (gl): Estadística de chi-cuadrado con grados de libertad, CFI: Comparative Fit Index, TLI: Tucker-Lewis Index, SRMR: Standardized Root Mean Square Residual, RMSEA: Root Mean Square Error of Approximation, Δ: difference.

En cuanto a la validez externa basada en la relación con otra variable se aprecia que el puntaje global del WAST Quechua Collao y la Patient Health Questionnaire (PHQ-9) presentan una relación directa, altamente significativa y una fuerza moderada de relación (Rho=0,618, p=0,001); frente a ello se confirma la validez externa. Asimismo, el modelo unidimensional del WAST quechua Collao reportó una buena confiabilidad presentando valores de alfa de Cronbach de 0,860 y omega McDonald de 0,867.

## DISCUSIÓN

En una muestra de 521 mujeres quechua hablantes bilingües, donde la mayoría tenían edades entre 18 a 34 años, secundaria completa, viven en el sector urbano y perciben mensualmente una economía familiar menor a 1,025 soles, el WAST quechua Collao con un modelo unidimensional presentó adecuados índices de bondad de ajuste que garantiza la validez de su estructura interna; por otro lado, la validez externa fue adecuada al relacionar las puntuaciones del WAST y PHQ-9 en sus versiones en quechua Collao, también presentó una confiabilidad óptima y fue invariante por grupos de edad, grado de instrucción, lugar de vivienda e ingreso económico familiar mensual.

El modelo unifactorial del WAST quechua Collao reportó adecuados ajustes, lo que sugiere que es un buen modelo para la evaluación de la violencia de pareja como un único factor. Los índices de ajuste, como el CFI (0,995) y el TLI (0,992), indican un excelente ajuste del modelo, ya que ambos valores están muy cerca de 1. El SRMR (0,051) también está dentro del parámetro de aceptabilidad (<0,08), lo cual sugiere una buena representación de los datos observados; mientras que el RMSEA (0,063) está por debajo del umbral de 0,08, indicando un ajuste razonable del modelo ^28^. Estos resultados en conjunto demuestran que el modelo de un solo factor del WAST quechua Collao es robusto y adecuado, lo que respalda su validez a nivel de estructura interna para medir la violencia de pareja en mujeres quechua hablantes. De hecho, estudios previos en mujeres jóvenes ^17^ y mujeres adultas ^18^^)^ también reportaron índices de bondad de ajuste similares confirmando que este instrumento es válido para la población peruana.

Nuestro estudio evidenció que el WAST quechua Collao presentó invarianza de medida entre los grupos definidos por edad, nivel educativo, lugar de residencia e ingreso económico familiar mensual. De forma similar, la versión en español del WAST validada en mujeres peruanas también mostró invarianza en función de la edad, nivel educativo, lugar de vivienda y situación laboral ^18^. Sin embargo, en el análisis de invarianza métrica por nivel de ingreso económico familiar mensual, se obtuvo un valor de RMSEA=0,090, ligeramente superior al umbral recomendado (<0,08). Este resultado podría estar influenciado por el tamaño desigual o reducido de algunas submuestras dentro de esta variable sociodemográfica, lo cual puede afectar la estabilidad de las estimaciones del modelo y aumentar artificialmente los índices de error de aproximación. Es importante señalar que el RMSEA es particularmente sensible a modelos con grados de libertad bajos o tamaños muestrales pequeños en subgrupos, lo que puede conducir a una sobreestimación del error de ajuste incluso en modelos correctamente especificados. A pesar de este valor ligeramente mayor, estudios de simulación han sugerido que valores de RMSEA hasta 0,10 pueden ser considerados aceptables en ciertas condiciones, especialmente cuando otros índices como el CFI se mantienen dentro de los rangos adecuados ^29^. Asimismo, el estudio previo con la versión en español del WAST en población peruana reportaron valores de RMSEA similares en sus análisis de invarianza ^18^, lo que refuerza la robustez del presente hallazgo.

El puntaje global del WAST Quechua Collao y de la Patient Health Questionnaire (PHQ-9) presentaron una relación directa, altamente significativa y una fuerza moderada de relación (Rho=0,618, p=0,001). Otro estudio también reportó resultados similares pues el WAST versión en español peruano reportó una relación con la puntuación del PHQ-9 (Rho=0,620; p=0,001). Este hallazgo indica que a medida que aumentan los puntajes del WAST Quechua Collao, también incrementan la puntuación del PHQ-9. Por otro lado, la fuerza de relación moderada (Rho=0,618) sugiere una asociación considerable entre las dos medidas y el valor p altamente significativo (p=0,001) confirma que la correlación observada es altamente improbable que ocurra por azar; de esta manera se confirma de manera sólida la validez de criterio o basada en la relación con otras variables, indicando que es una herramienta válida para evaluar la violencia de pareja en contextos clínicos y de investigación. La razón por la que se determinó relacionar con una prueba que evalúa la depresión para evaluar la validez externa, fue debido a la fuerte asociación entre violencia de pareja y síntomas depresivos documentada en diversos estudios ^30^^,^^31^.

El WAST quechua Collao reportó una buena confiabilidad, con valores de alfa de Cronbach de 0,860 y Omega McDonald de 0,867. Estos hallazgos fueron similares al encontrado en la versión peruana para mujeres adultas hispanohablantes donde los coeficientes de fiabilidad α = 0,899 y ω = 0,916 también reportaron óptimos valores ^18^. Aquello, quiere indicar que el WAST quechua Collao, presenta una sólida evidencia de precisión y consistencia en la medición en diferentes momentos para esta población. Dado que la fiabilidad se entiende como una característica de las puntuaciones de un instrumento que analiza la precisión de las mediciones en diferentes momentos y relacionado principalmente al error aleatorio donde a mayor error aleatorio menor la confiabilidad; a pesar del cuestionamiento del uso del alfa de Cronbach por presentar limitaciones a partir del número de ítems, alternativas de respuesta y la proporción de varianza ^26^ presentó un coeficiente dentro del parámetro de óptima confiabilidad, tal y como lo hizo el coeficiente omega de McDonald’s que a diferencia del alfa clásico, estima la fiabilidad a partir de las cargas factoriales que hace más estable los cálculos y refleja un real valor de fiabilidad ^32^.

Los ítems del WAST son empleados en el tamizaje de salud mental en la atención primaria de la salud y programas del Estado identificar tempranamente los indicadores de violencia de pareja. El WAST quechua Collao podría ser implementado en este sistema para poblaciones quechua hablantes de la variedad Collao hablado en el sur peruano. Por lo que las evaluaciones futuras podrían tener un mejor acercamiento al problema real de la violencia de pareja en poblaciones originarias.

El estudio presenta varias limitaciones, como el hecho de que está validada en mujeres con escolaridad y saben leer en quechua, por lo que mujeres que no cuentan con escolaridad no podrían ser evaluadas con esta versión escrita, estudios posteriores podrían diseñar una versión en audio que logre llegar a estas poblaciones no escolarizadas. Por otro lado, aunque se observaron correlaciones significativas entre el PHQ-9 y WAST en quechua Collao, estas no deben interpretarse como evidencia de causalidad; puesto que, nuestro instrumento puede presentar alta validez externa en esta población específica, pero no necesariamente en otros. Por ello, se recomienda cautela al generalizar estos hallazgos a otras poblaciones quechua hablantes o en contextos culturales distintos. Otra de las limitaciones es la posible presencia de sesgo por deseabilidad social, lo cual limita la precisión en la detección de casos reales; aunque se garantizó el anonimato, no se descarta que algunas participantes hayan subestimado su experiencia con la violencia de pareja debido a normas culturales o temor al estigma. Además, otra limitación relevante es que el WAST quechua Collao evalúa la violencia de pareja desde una perspectiva unidimensional; aunque esta característica permite una detección rápida y general del problema, también limita su capacidad para identificar con precisión la naturaleza específica de la violencia experimentada. En consecuencia, si bien el instrumento resulta útil para el tamizaje inicial, no reemplaza evaluaciones más detalladas por profesionales pertinentes que permitan caracterizar los distintos tipos de violencia. La principal fortaleza de nuestro estudio es que se trata del primer instrumento de violencia de pareja adaptado al contexto cultural del quechua Collao que servirá para poder identificar tempranamente y de manera más precisa los casos de violencia de pareja desatendida por la brecha cultural y lingüística.

En conclusión, el WAST en quechua Collao de un modelo unidimensional presentó validez de su estructura interna, validez externa, una óptima fiabilidad y resultó ser invariante por grupos de edad, grado de instrucción, lugar de vivienda e ingreso económico familiar mensual en mujeres peruanas; frente a ello, su uso es recomendado para identificar de forma temprana los casos de violencia de pareja en mujeres que hablan quechua Collao.
